# Medical Offenders

**Published:** 1899-03-18

**Authors:** 


### MEDICAL OFFENDERS.

THE President and the legal advisers of the General Medical Council have been engaged, now for a considerable period of time, in a praiseworthy endeavour to unite the various licensing authorities of the medical profession in an endeavour to establish some common principle for dealing with offenders against the 29th clause of the Medical Act, by which the Council is able to erase from the Register the names of persons convicted of conduct "infamous in a professional respect." For some of the offences thus described it has been felt that permanent loss of professional status was too severe a punishment; and so the Council, acting upon the common law right of a corporation to rescind its own acts, but without any special statutory authority, has in several instances directed the restoration of the names of such persons, after a period of two or three years from the erasure. The licensing corporations, as a rule, follow the original action of the Council, and erase the names from their own lists also, but some of them have no power to restore without subjecting the offender to another examination; while the universities, as a rule, either cannot or will not do anything, with the result that an offender who has ceased to be on the Medical Register, and has ceased in consequence to be either a licentiate of the Royal College of Physicians or of the Society of Apothecaries, or a Member of the College of Surgeons, may still be a Doctor of Medicine of a distinguished university, and may be able to use the title of his degree on all occasions. The Privy Council would not be opposed to the introduction of a Bill by which this anomaly would be removed; but it is difficult to frame the clauses in such a way as to meet the views of all the bodies concerned, and there seems no probability that any effective action can be taken during the present session of Parliament. The probable result will be a compromise, under which every licensing body will receive powers to follow the action of the Council if they see fit, and either to remove or to restore their graduates, members, or licentiates, in any circumstances which render the use of such powers expedient.

